# Real-time beat-to-beat pulse wave velocity estimation: a quality-driven approach using laser Doppler vibrometry

**DOI:** 10.1007/s11517-025-03417-8

**Published:** 2025-07-30

**Authors:** Silvia Seoni, Patrick Segers, Simeon Beeckman, Massimo Salvi, Marco Romanelli, Smriti Badhwar, Rosa Maria Bruno, Yanlu Li, Soren Aasmul, Nilesh Madhu, Filippo Molinari, Umberto Morbiducci

**Affiliations:** 1 Biolab, PoliTo BIOMed Lab, Department of Electronics and Telecommunications, Politecnico di Torino, Corso Duca degli Abruzzi 24, 10129 Turin, Italy; 2 BioMMeda – Institute for Biomedical Engineering and Technology, Ghent University, Ghent, Belgium; 3 Laboratory of Integrative Physiology, Department of Neuroscience, Università di Torino, c.so Raffaello 30, Torino, 10125 Italy; 4 Paris Centre de Recherche Cardiovasculaire, INSERM, Paris, France; 5 Assistance Publique Hôpitaux de Paris, Hôpital Européen Georges Pompidou, Paris, France; 6 Photonic Research Group, Ghent University-IMEC, Technologiepark-Zwijnaarde 126, Ghent, 9052 Belgium; 7 Center for Nano- and Biophotonics, Ghent University, Technologiepark-Zwijnaarde 126, Ghent, 9052 Belgium; 8 arly Feasibility and Sensor Technology, Medtronic Bakken Research Center, Maastricht, Netherlands; 9 IDLab – Internet and Data Science Lab, Ghent University – imec, Ghent, Belgium; 10 Department of Mechanical and Aerospace Engineering, PolitoBIOMed Lab, Politecnico di Torino, Corso Duca degli Abruzzi, Torino 24, Turin , 10129 Italy

**Keywords:** Pulse wave velocity, Laser Doppler vibrometer, Real-time PWV estimation, Quality assessment

## Abstract

Arterial stiffness, a key cardiovascular risk marker, is typically assessed via carotid-femoral pulse wave velocity (cf-PWV), the gold-standard method. In this study, we introduce CAPE (Continuous Automatic PWV Estimation), an innovative framework for near real-time cf-PWV estimation based on beat-to-beat analysis of laser-Doppler vibrometry (LDV) signals. CAPE integrates automatic fiducial point detection, systematic signal quality control, and a cross-channel strategy to provide a highly reliable assessment of cf-PWV. The framework was evaluated using LDV signals acquired from 100 patients with mild to moderate essential hypertension, using a multichannel laser vibrometry system. CAPE calculates cf-PWV as the ratio of carotid-femoral distance to pulse transit time (PTT), which is the delay between carotid and femoral fiducial points. These points are detected using template-matching on the second derivative of LDV displacement signals. Signal quality in CAPE is ensured through an integrated quality assessment based on the number of automatically detected carotid-femoral peaks, which assigns confidence scores (acceptable or excellent) to the PWV measurements. When validated against the gold-standard applanation tonometry, CAPE achieved a mean bias of 0.25 ± 0.77 m/s, demonstrating high reliability and precision. The optimized framework estimates cf-PWV in 3 s, making CAPE ideal for clinical applications requiring real-time cardiovascular assessment.

**Graphical abstract:**

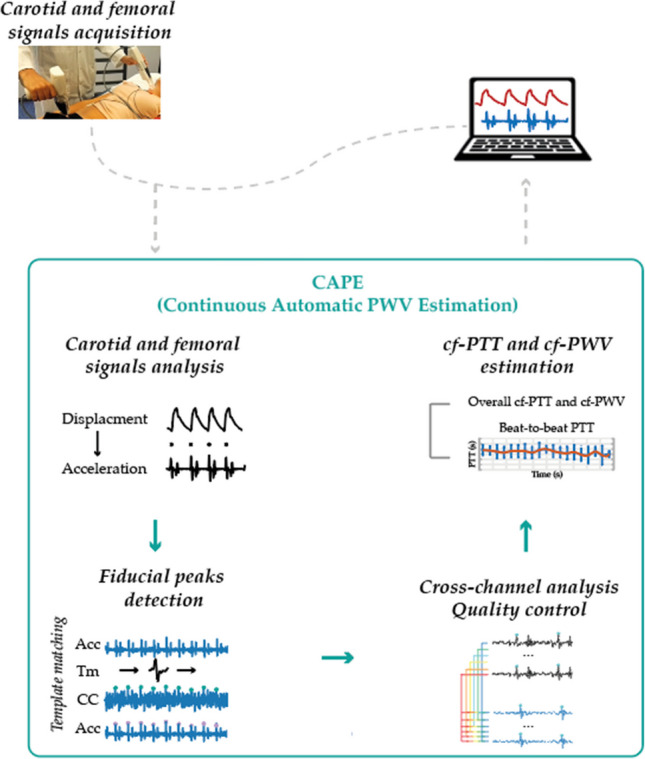

## Introduction

The aorta and large central arteries buffer the heart’s pulsatile output by expanding with each beat to store elastic energy, which is released during relaxation to maintain continuous blood flow and stable pressure [1, 2]. With age or disease, arterial stiffening reduces the buffering capacity of arteries, increasing the risk of, e.g. stroke, cardiac failure, chronic renal disease and end-organ damage [3]. Based on evidence, over the past few decades, arterial stiffness has been increasingly recognized as a key indicator of cardiovascular risk and mortality [4, 5].

Arterial stiffness can be evaluated by measuring the so-called pulse wave velocity (PWV) which refers to the speed at which pressure waves propagate through the arterial tree during the systolic contraction. High PWV values indicate reduced vessel distensibility, reflecting greater arterial stiffness [6]. PWV is typically measured between two arterial sites, one proximal and one distal, to assess the velocity along a segment of the arterial pathway of defined length. In this regard, carotid-femoral PWV is considered the gold-standard method for assessing arterial stiffness, where pressure waveforms are captured from the carotid and femoral arteries, located in the neck and groin, respectively [5]. PWV is determined by the ratio of the distance between two arterial sites, to the transit time of the pulse wave between the two measurement sites.

A non-invasive, commonly adopted technique for measuring PWV is applanation tonometry. Various algorithms can be implemented within applanation tonometry to enhance the accuracy of PWV measurements. These algorithms have been developed to improve the accuracy of fiducial point detection and pulse transit time (PTT) estimation, with the foot of the waveform often serving as the primary reference point [7, 8]. Foot-to-foot methods such as the diastole-minimum, maximum first derivative, maximum second derivative, and tangential methods are frequently employed, along with region-based approaches like the diastole-patching method [9] or region-based cross-correlation approach [10]. However, current automated PWV estimation methods often face challenges including operator dependency, sensitivity to signal noise, and lack of real-time quality assessment capabilities. These limitations can lead to unreliable measurements and require multiple recording attempts in clinical settings.

In some of the proposed implementations, applanation tonometry is combined with ECG signals, where the R-wave of the ECG provides a timing reference to allow for sequential measurements at the two measuring sites [11, 12]. Other technologies, such as ultrasound, magnetic resonance imaging (MRI), and accelerometers, are also used to detect arterial pulses and estimate PWV [3, 13, 14]. Ultrasound-based devices, for instance, rely on Doppler signals or vessel wall motion tracking to measure PTT [15, 16]. Regional aortic stiffness can be measured using 4-dimensional flow cardiac magnetic resonance [17, 18]. However, non-invasive PWV measurements present practical challenges hampering their large adoption [3], which have motivated the exploration of alternative methods. These practical limitations highlighted the need for more accessible and operator-independent measurement techniques. Laser-Doppler vibrometry (LDV) has emerged as a promising non-contact technique, detecting skin motion overlying the carotid and femoral arteries and offering a viable method for PWV estimation [19–22]. There is a large body of literature confirming that LDV offers a simple, noninvasive and operator-independent method for assessing arterial stiffness, with results comparable to established methods [19–22]. In previous studies, the feasibility of LDV for PWV measurements was demonstrated using industrial-grade sensors [20]. As part of the H2020-funded CARDIS project, a multi-beam handheld device was developed, incorporating a silicon photonics chip into a compact design, enabling flexible, multi-array configurations [23]. The prototype consists of two separable handheld units for simultaneous measurement of the neck and groin, each equipped with six channels with a laser beam. In a clinical feasibility study, carotid-femoral PWV (cf-PWV) measurements from this device were compared to a reference method using applanation tonometry, yielding promising results [24]. Furthermore, Badhwar et al. [25] demonstrated the clinical validity of LDV-based measurements of carotid-femoral PWV (cf-PWV) compared to standard reference techniques.

This study presents CAPE (Continuous Automatic PWV Estimation), a near real-time, accuracy-controlled framework for cf-PWV estimation using an LDV system. CAPE offers a continuous (beat-to-beat analysis) as well as an overall cf-PWV estimation, providing a comprehensive understanding of cardiovascular dynamics. The approach builds upon our previously validated methodology for automatic fiducial point detection and signal quality assessment, thoroughly described in [26], which is now embedded within CAPE to enable robust and confidence-classified cf-PWV measurements. The main contributions of this paper are as follows:

- A near real-time automatic algorithm for cf-PWV estimation using an LDV system (with results available within approximately 3 s after signal acquisition), enabling continuous cardiovascular monitoring with minimal operator intervention and rapid clinical feedback
- The integration of automatic fiducial point detection with a quality criterion, ensuring accurate and reliable cf-PWV estimation with systematic signal quality assessment
- An LDV multichannel acquisition implementing a cross-channel strategy and an innovative beat-to-beat analysis for cf-PWV estimation
- Based on signal quality, a quality-controlled algorithm with integrated confidence level assessment for cf-PWV measurement
- This paper is structured as follows: Sect. 2 provides a comprehensive overview of the proposed method, while Sect. 3 details the experimental results. Finally, Sects. 4 and 5 offer a thorough discussion of the overall work.

## Materials and methods

### Dataset

The dataset used in this study was acquired as part of the CARDIS project [24]. The CARDIS project recruited 100 patients, both male and female, aged between 18 and 85 years, diagnosed with mild to moderate essential hypertension (systolic blood pressure ranging from 140 to 179 mm Hg and diastolic blood pressure ranging from 90 to 109 mm Hg). The study was conducted at the Georges Pompidou European Hospital (Paris, France), where patients referred by the Hypertension and Pharmacology units underwent carotid-femoral pulse wave velocity (cf-PWV) assessment in the vascular laboratory as part of routine clinical care.

To ensure stable physiological conditions, all measurements were performed in the supine position following a 10-min rest period, in accordance with the European Society of Hypertension (ESH-ESC 2018 guidelines) [27]. Three consecutive measurements of blood pressure and heart rate were obtained using a validated oscillometric device (Colin Press-Mate BP monitor) immediately before cf-PWV acquisition.

The applanation tonometry measurements were acquired first using the SphygmoCor system (Atcor Medical, Australia), which served as the reference method in this study. On the same day and within the same clinical session, LDV signals were recorded using the CARDIS device from the carotid and femoral arteries. Although the two measurements were not simultaneous, the temporal gap was minimal, and both were acquired under comparable resting conditions to limit potential hemodynamic variability.

The LDV signals used in this study were recorded in an ambulatory setting, replicating real-world conditions, allowing for the evaluation of CAPE’s performance under conditions where motion artifacts might be more prevalent.

Exclusion criteria included secondary hypertension, established cardiovascular diseases such as a history of acute heart failure, unstable coronary heart disease, peripheral arterial disease, stroke and arrhythmias. Additionally, patients with chronic inflammatory or infectious diseases were excluded from the study. The study received approval from the National Ethics Committee (Comité de Protection des Personnes) and is registered under ClinicalTrials.gov with ID: NCT03446430.

Raw LDV data in the form of in-phase and quadrature (IQ) signals were acquired at a sampling frequency of 100 kHz. The resulting LDV displacement signals were downsampled to 10 kHz upon demodulation. An IIR infinite impulse response low-pass filter with a cut-off frequency of 30 Hz was applied to LDV displacement recorded signals, which were then differentiated twice to obtain acceleration. Note that this filtering was also applied after *each* differentiation step to further suppress high-frequency noise. The filtering was performed using zero-phase forward–backward filtering to avoid phase distortion and preserve the temporal integrity of the signal features.

### LDV device

**Fig. 1:**
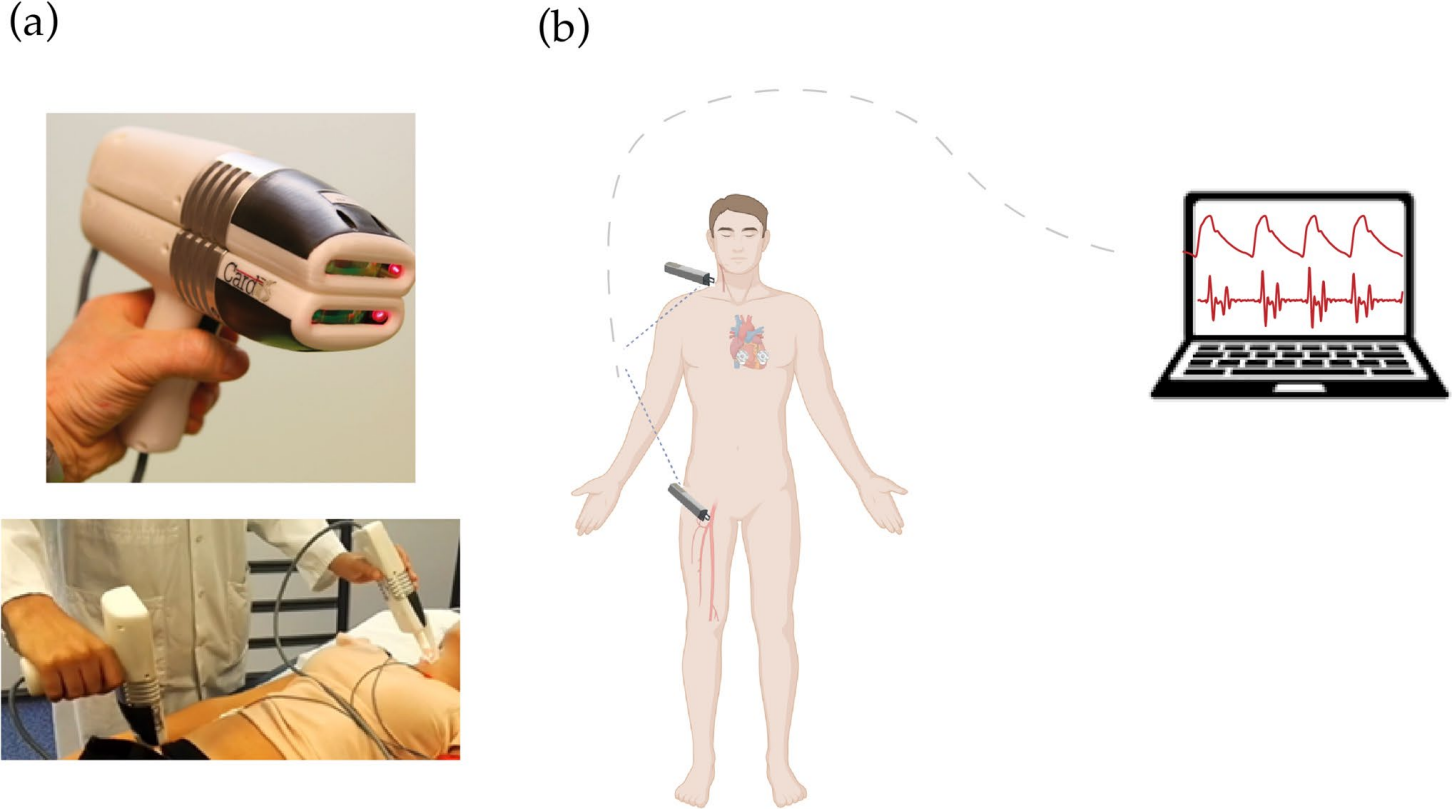
Cardis LDV device: illustration of the two handpieces used in either connected or separate configuration for a carotid–femoral acquisition (**a**), and schematic of the measurement setup (**b**)

An extensive description of the CARDIS device and the embedded optical system is provided in Li et al. [23]. Briefly, the device comprises two main components: handpiece 1, which includes the primary grip, and handpiece 2, an auxiliary extension. The primary handpiece was placed at the femoral artery and the secondary handpiece at the carotid artery for simultaneous measurement from the two arterial sites for carotid-femoral PWV measurements. Each handpiece projects a series of six laser beams (wavelength 1550 nm) arranged linearly with a 5-mm interval between them. To enhance the reflection of the laser beams, a retro-reflective tape is applied to the measurement area on the skin. Additionally, the device includes a spacer to ensure optimal optical focus and stability during measurements. Figure 1 displays an illustration of the device and the positioning of these handpieces (a) and a measurements setup (b). A single measurement consists of six LDV signals acquired at the carotid site and six LDV signals acquired at the femoral site.

### Automatic algorithm for PTT and PWV estimation

**Fig. 2:**
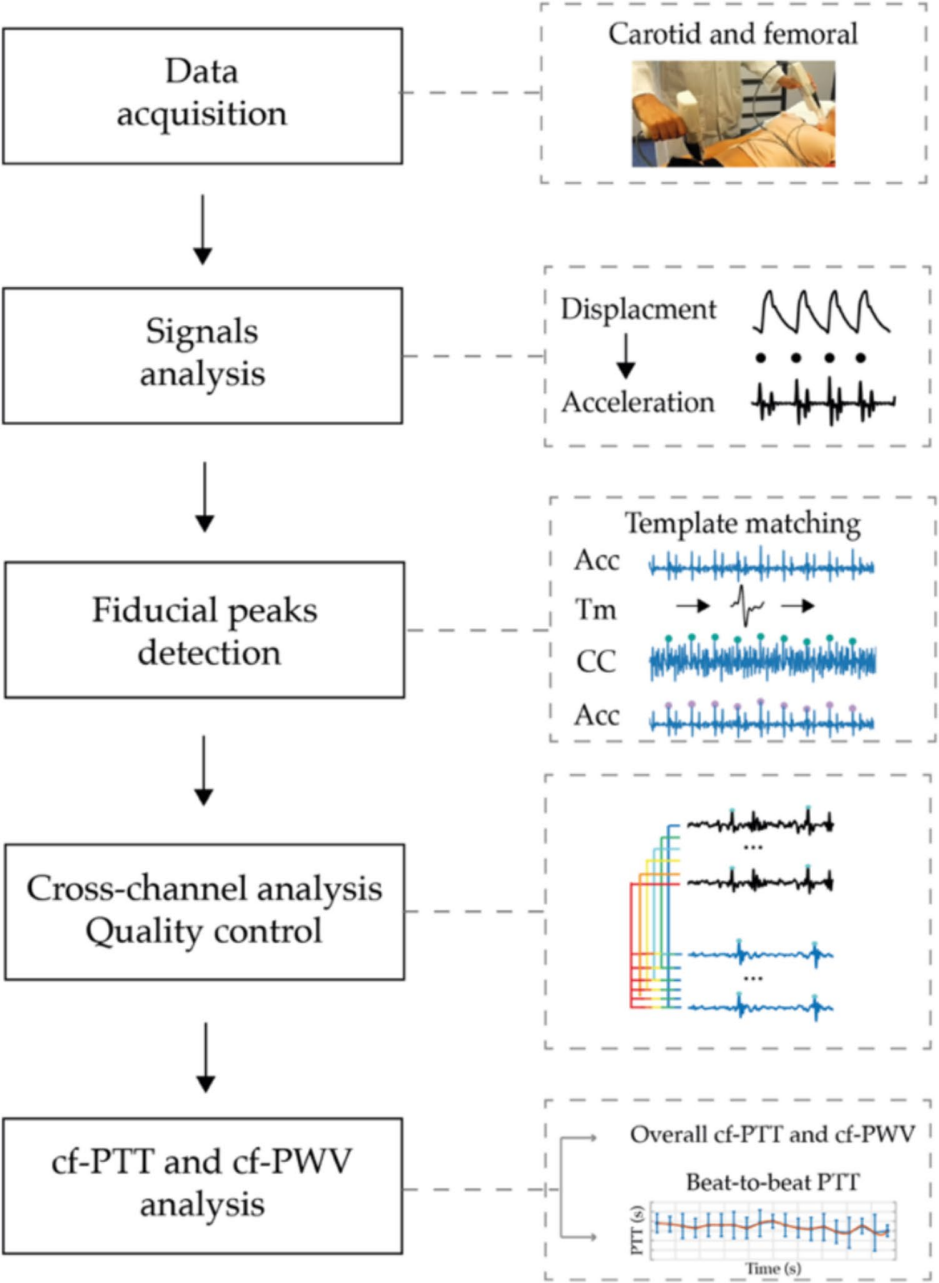
CAPE framework: starting with the displacement signals acquired by the CARDIS device, the CAPE algorithm calculates the second derivative (acceleration signals) and automatically identifies the fiducial points as the peaks of these acceleration signals. Following a cross-analysis of all cf-peak pairs and a quality control check, CAPE computes cf-PTT values. This process provides an overall PTT and cf-PWV (carotid-femoral pulse wave velocity) value, along with a trend analysis of PTT values over time. In the figure, ACC, TM, CC, and cf represent the acceleration, the template, the cross-correlation function, and the carotid-femoral

An overview of the CAPE framework for the cf-PWV analysis is presented in Fig. 2. LDV signals are acquired using the CARDIS device from femoral and carotid sites. The panel of Fig. 2 displays an example of LDV recording from one of the six channels (in terms of displacement signal) at the carotid site together with its second derivative (acceleration signal), the latter being used to identify the fiducial points for the PTT estimation. In detail, in the CAPE framework, the cf-PTT is defined as the time delay between the maximum of the second derivative (fiducial points) of the LDV displacement signals acquired at the carotid and femoral artery measurement sites. The adoption of the second derivative data, corresponding to skin acceleration, minimizes motion drift and enhances sensitivity to rapid changes associated with the pulse wave’s arrival [28]. The second derivative was specifically chosen as it amplifies the sharp features of the waveform while suppressing low-frequency motion artifacts, making it particularly suitable for accurate fiducial point detection. This approach has been shown to be more robust than using either the raw displacement signal or first derivative, especially in cases where baseline drift or patient movement might affect the measurements.

Initially, template matching is applied to accurately identify the fiducial points on the acceleration data [26]. Considering the six LDV signals acquired from each of the two handpieces (Fig. 1), the proposed algorithm performs a cross-channel analysis of all the possible combinations of carotid-femoral peak pairs, resulting in 36 possible combinations per heartbeat. The CAPE algorithm aggregates these cf-peak pairs for each heartbeat, effectively capturing a comprehensive set of peak pairs that serves as a basis for PTT estimation. To ensure reliable PTT measurements [29], a quality criterion is then applied to individual heartbeats as well as to the overall recording, aiming to preserve the accuracy of PTT measurements. Technically, for each cf-peak pair, the CAPE algorithm calculates the PTT by measuring the delay between carotid and femoral acceleration peaks. The PTT values from each possible combination are then aggregated to perform a beat-by-beat analysis, producing a single PTT value for each heartbeat. An overall PTT value is computed by averaging the beat-by-beat PTT estimates, resulting in a comprehensive cf-PWV measurement and an associated confidence level based on signal quality. Specifically, this confidence level is classified as acceptable or excellent, according to threshold values defined by guidelines criteria [29].

#### Identification of the fiducial points

In the CAPE framework, the template matching algorithm is employed to detect peaks in the LDV acceleration signals according to the strategy described in our previous study [26]. This algorithm identifies acceleration waveforms that closely match a pre-constructed template [30, 31]. For this study, two templates were used: one for the carotid site and one for the femoral site. These templates were generated by averaging LDV epochs from high-quality signal recordings. High-quality signals were defined using an expert-based visual scoring system, where only those rated as excellent (score = 5) were included. Details on the template generation can be found in [26]. Each template spans 200 ms centered around the peak of the LDV acceleration signal. In our previous work [26], we evaluated multiple template durations and found that 200 ms offered the best performance for accurately detecting high-quality LDV acceleration peaks. Building on the previous study, here, we revised and expanded the approach to make the method more effective for clinical use and to enhance computational efficiency. Specifically, here, the cross-correlation function was employed to quantify the similarity between the template and LDV signals.

**Fig. 3:**
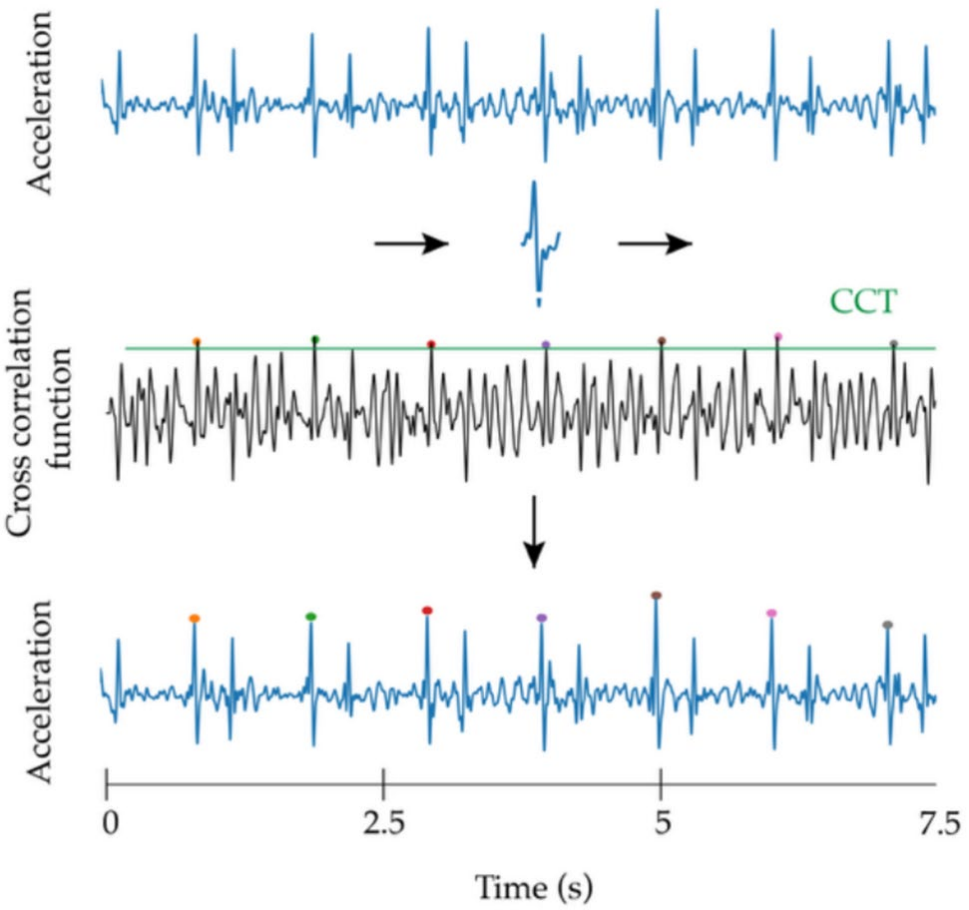
Fiducial peaks detection using the template matching algorithm. The template moves among the signals, and the cross-correlation function is computed (third line). The last line displays the acceleration signals with the detected peaks that correspond to the cross-correlation values higher than the cross-correlation threshold (CCT, green line in the figure). In this example, *K* is equal to 2.5. The template has a duration of 200 ms

To detect fiducial points within the LDV signals, a threshold level based on the cross-correlation amplitude, referred to as the cross-correlation threshold ($\text{CCT}$), was established according to

1 $$CCT =K\frac{1}{N}{\sum }_{i=1}^{N}\left|{r}_{x,y} [i]\right|$$

where subscript $x$ and $y$ denote the LDV signal and the template, respectively, and $N$ is the number of steps over which the cross-correlation is computed. Since the template is shorter than the LDV signal, it slides over the entire signal, with the cross-correlation calculated at each step. Consequently, $N$ represents the total number of cross-correlation operations performed as the template slides over the LDV signal. A fiducial point is detected when the cross-correlation value is higher than $\text{CCT}$. The sensitivity and specificity of the peak detection process are controlled by the tuning factor $K$, which directly influences PTT estimation accuracy. An example of the way the fiducial peaks detection and the template matching algorithms operate is displayed in Fig. 3.

The acquired signals from all 12 channels (6 from the carotid hand piece and 6 from the femoral handpiece) are processed simultaneously, using separate templates for carotid and femoral LDV signals while maintaining the same $K$ value for $\text{CCT}$ calculations. In this configuration, the algorithm can detect up to 36 cf peak pairs per heartbeat, based on the possible combinations of six carotid peaks and six femoral peaks. CAPE then aggregates the cf-peak pairs for each heartbeat, effectively capturing a comprehensive set of peak pairs for a detailed analysis of the specific heartbeat.

#### Automatic quality control

**Fig. 4:**
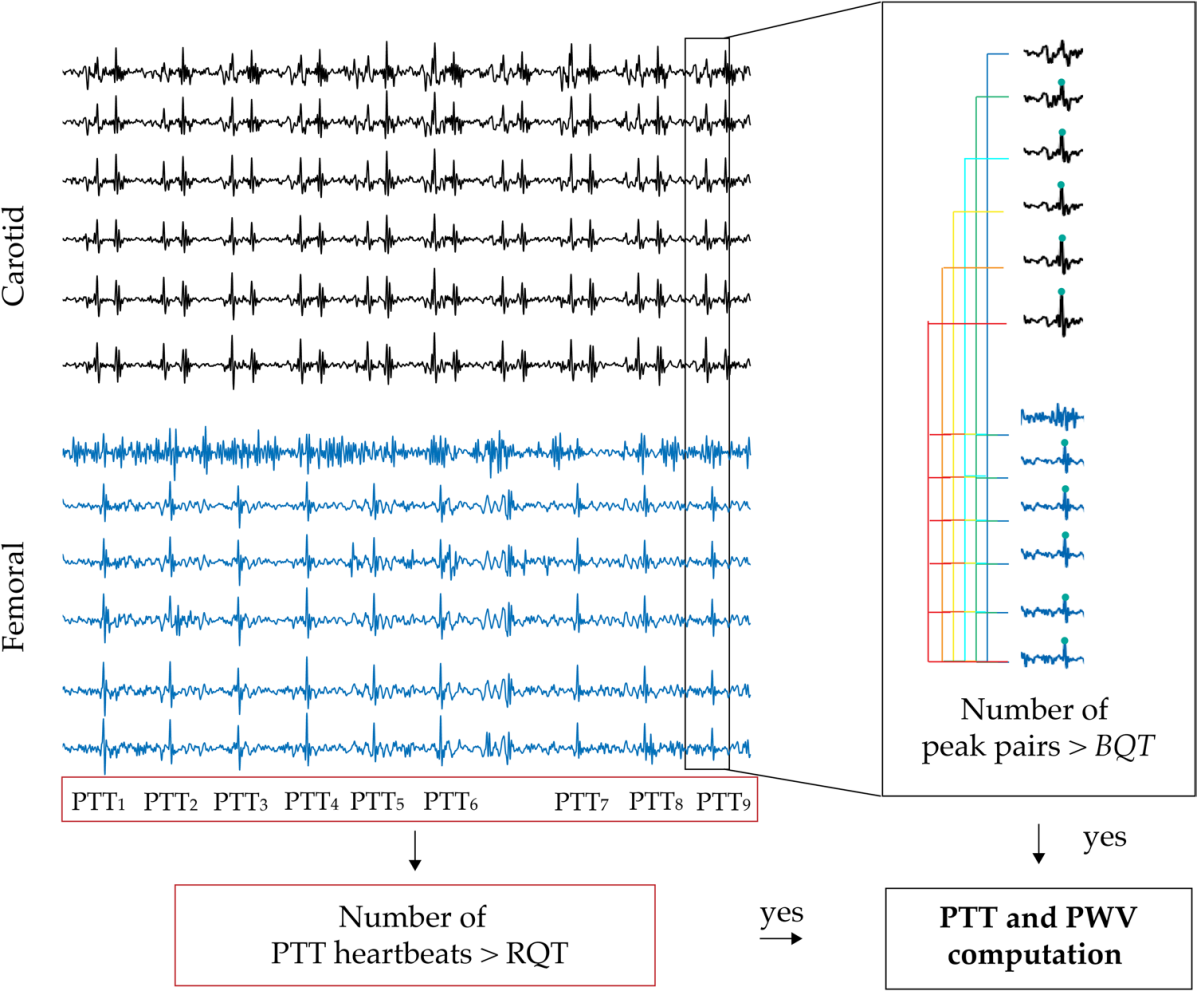
Quality criterion for the cf-PWV estimation. The black box represents the first module on the number of peak pairs (Beat Quality Threshold, BQT), while the red box is the second module on the number of good heartbeats (Recording Quality Threshold, RQT)

As already stated, signal quality is crucial for accurate PTT estimation. To ensure reliable measurements, we introduced a quality control criterion aimed at discarding low-quality and incorrectly detected peaks that could compromise the accuracy of PTT calculations. An example of the LDV acceleration signals acquired from each one of the two handpieces, where the two modules of quality criterion assessment are applied, is presented in Fig. 4. The quality assessment framework operates at two different levels: individual heartbeat or signal segment evaluation (*local*) and overall recording quality evaluation (*global*). This *local–global* approach is founded on the previous finding that the LDV acceleration signal quality can be effectively quantified through the number of accurately detected fiducial points [26].

At the first *local* quality control level, the beat quality threshold (*BQT*) module, defining the minimum number of carotid-femoral peak pairs (cf-peak pairs) required to classify a heartbeat as reliable for its use in delay estimation, is applied. If the number of detected cf-peak pairs is lower than *BQT*, the heartbeat is discarded since the PTT estimation may lack accuracy and could lead to unreliable results. Conversely, if the number of identified peak pairs within a heartbeat is higher than *BQT*, the estimation of the delay becomes more robust, increasing the likelihood of accurate PTT estimates.

The second *global* quality control level implements the recording quality threshold (*RQT*) module, which establishes the minimum number of high-quality heartbeats required for a recording to be considered valid. This ensures that in recordings with an insufficient number of high-quality heartbeats, the algorithm will not provide any estimate of cf-PWV, as the entire acquisition is considered unaffordable for PTT estimation. The tuning of the threshold values for signal quality selection was conducted in accordance with international guidelines [29], ensuring that the algorithm meets the required standards for accuracy and reliability.

#### PTT estimation

**Fig. 5:**
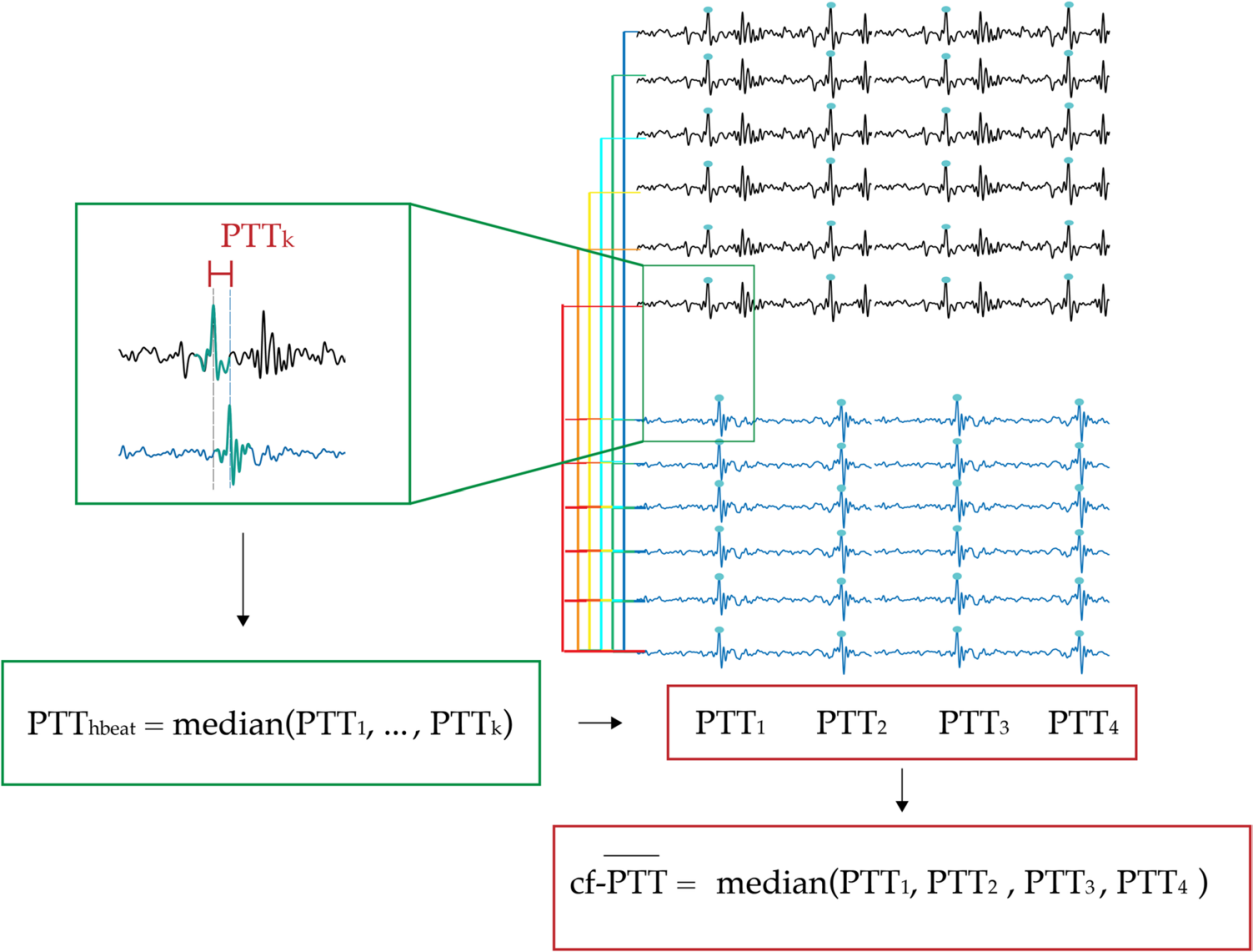
Automatic estimation of final cf-PTT: CAPE first computes the pulse transit time (PTT) between all peak pairs (green square) within each individual heartbeat (indicated by the green rectangle). It then determines the final PTT value by taking the median of the PTT values across all heartbeats (indicated by the red rectangle)

The cf-PWV estimation is based on a three-step process of delay computation. Figure 5 illustrates an example of a combination of cf-peak pairs used in the cf-PWV estimation process. First, for each cf-peak pair, the algorithm calculates the PTT as the temporal distance between the two acceleration cf-peaks (green square). Then, if a heartbeat presents a higher number of cf-peak pairs than *BQT*, the algorithm computes the median value of all delays of the corresponding heartbeat (${\text{PTT}}_{\text{hbeat}}$). In this way, heartbeats that do not meet the BQT threshold are excluded from the estimation. Finally, when the number of valid heartbeats is higher than the *RQT*, the algorithm computes the overall PTT for the entire recording, denoted $\overline{PTT },$ in terms of median value of all ${\text{PTT}}_{\text{hbeat}}$ values.

**Fig. 6:**
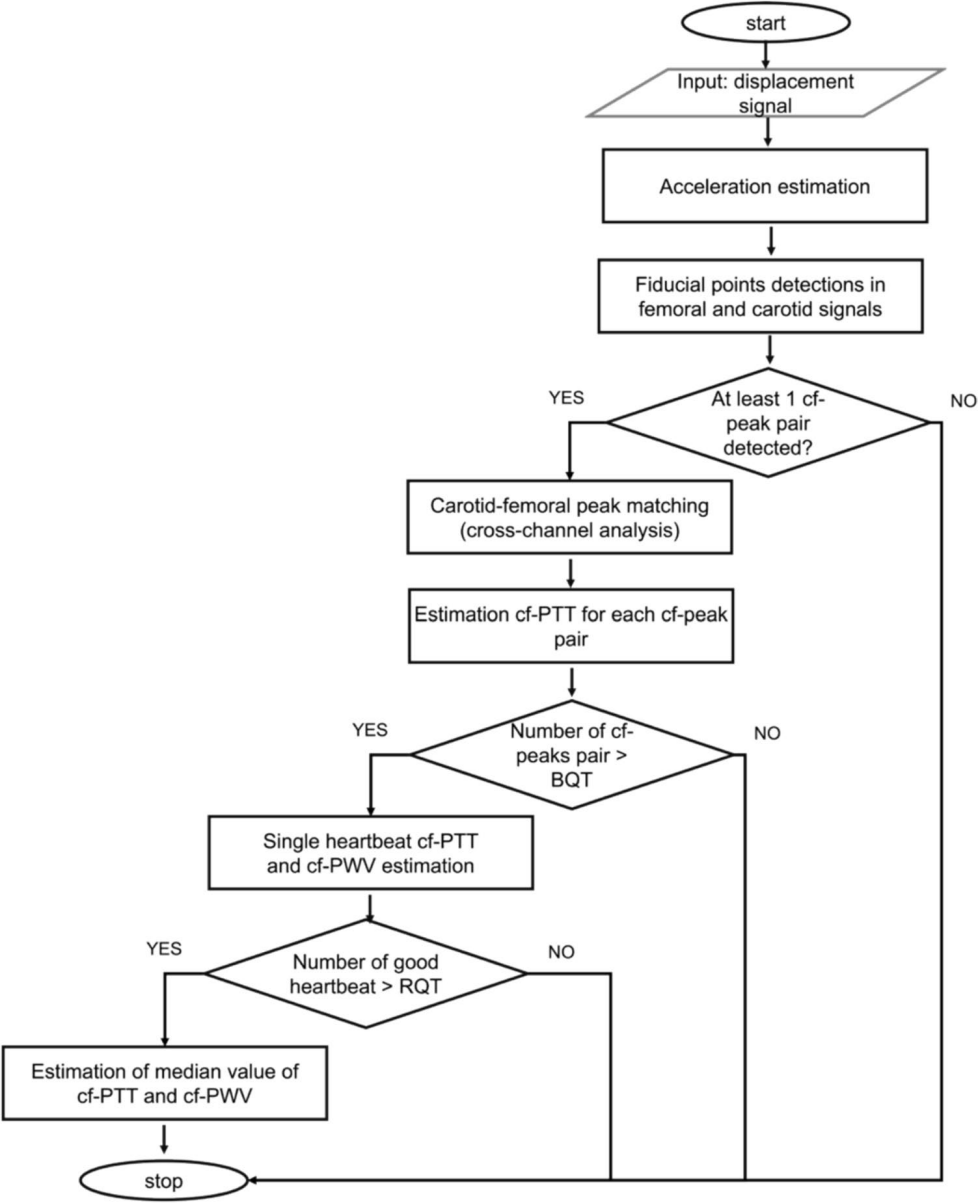
A comprehensive overview of the CAPE framework

As recommended by the expert consensus document on the measurement of aortic stiffness [29], the final cf-PWV is computed as follows:

2 $$\text{PWV }=0.8*d/\text{PTT}$$

where $d$ is the directly measured straight distance between the carotid and femoral sites, $\text{PTT}$ is the computed delay, and 0.8 is a correction factor [29]. The proposed algorithm offers two distinct analyses: an overall cf-PWV estimation over the entire recording, and a time-based assessment of cf-PWV variation, providing a detailed beat-to-beat evaluation of cf-PWV trends. A final comprehensive overview of the CAPE framework is displayed in Fig. 6.

### Validation and ablation analysis

#### Validation of cf-PWV estimation

The automatic algorithm for cf-PWV estimation was validated against applanation tonometry measurements (SphygmoCor System) [32], considered as the gold standard. The equivalence of the applanation tonometry-based and of the LDV-based approaches was tested through Bland–Altman analysis [33, 34], calculating the mean differences between the two estimated cf-PWV data, along with their standard deviations. Following international guidelines criteria [29], two cf-PWV measurements are considered equivalent if the mean difference is less than 0.5 m/s with a standard deviation (SD) below 0.8 m/s.

Based on guidelines, measurements are classified into three quality grades:

- Excellent: mean difference < 0.5 m/s and SD < 0.8 m/s
- Acceptable: mean difference < 1.0 m/s and SD < 1.5 m/s
- Poor: mean difference > 1.0 m/s or SD > 1.5 m/s

To optimize the PWV estimation, a systematic tuning and ablation analysis was conducted for three key variables (*K*, BQT, and RQT) of the CAPE framework:

- The value of *K* was varied from 1 to 5, in increments of 0.5.
- The value of *BQT* was varied from 10 to 20, in increments of 5.
- The value of *RQT* was varied from 5 to 15, in increments of 5, considering a recording duration of 20 s.

#### Confidence level of the cf-PWV estimation

The CAPE framework, implementing an adaptive validation approach through dynamic adjustment of BQT and RQT thresholds, enables quality-dependent accuracy levels. This flexibility allows the algorithm to operate effectively across varying signal quality conditions, with three distinct response modes:

- Low-quality signals: The algorithm automatically withholds PWV estimation when signal quality falls below minimum thresholds, preventing unreliable measurements.
- Moderate-quality signals: For signals meeting basic quality measurements, the algorithm provides acceptable PTT estimates, ensuring usable measurements while acknowledging potential limitations in precision.
- High-quality signals: When the signal quality is high, the algorithm delivers an excellent measurement with high-accuracy PTT estimates.

Following established guidelines [29], two distinct threshold configurations were implemented to optimize the algorithm performance. The first configuration focuses on high-precision measurements, optimized specifically for high-quality signals to deliver maximum accuracy in PTT estimation. The second configuration adapts to moderate-quality signals, providing acceptable measurement precision while maintaining reliability. This dual-configuration approach ensures the algorithm’s versatility across varying signal quality conditions while maintaining measurement reliability.

#### Ablation analysis

To assess the impact of various quality control modules on cf-PWV estimation, an ablation analysis was performed by systematically disabling specific thresholds. Disabling the BQT threshold allowed for the evaluation of how single-beat quality control influences the overall accuracy of cf-PWV estimation. Similarly, disabling the RQT threshold assessed the effect of quality control applied to the entire recording on the final cf-PWV estimate.

Additionally, we evaluated the computation of the PTT by implementing four distinct statistical approaches, applied to both individual beats and the entire recording:

3 $${\text{PTT}}_{\text{mean}\_\text{mean}}= \frac{1}{N}\sum_{i=1}^{N}\left(\frac{1}{{k}_{i}}\sum_{j=1}^{{k}_{1}}{\text{PTT}}_{h\text{beat},1, j} , \dots , \frac{1}{{k}_{N}}\sum_{j=1}^{{k}_{N}}{\text{PTT}}_{h\text{beat},N, j}\right)$$

4 $${\text{PTT}}_{\text{mean}\_\text{median}}= \frac{1}{N}\sum_{i=1}^{N}\left({\text{Median}(\text{PTT}}_{h\text{beat}, 1}\right), \dots , {\text{Median}(\text{PTT}}_{h\text{beat},\text{ N}}))$$

5 $${\text{PTT}}_{\text{mean}\_\text{mean}}= \text{Median}\left(\frac{1}{k}\sum_{j=1}^{k}{\text{PTT}}_{h\text{beat},1, j} , \dots , \frac{1}{{k}_{N}}\sum_{j=1}^{{k}_{N}}{\text{PTT}}_{h\text{beat},N, j}\right)$$

6 $${\text{PTT}}_{\text{median}\_\text{median}}= \text{Median}\left({\text{Median}(\text{PTT}}_{h\text{beat}, 1}\right), \dots , {\text{Median}(\text{PTT}}_{\text{hbeat}, N}))$$

where ${\varvec{N}}$ represents the number of heartbeat and ${\varvec{k}}$ the number of carotid-femoral pair in the specific heartbeat.

## Results

### Validation of cf-PWV estimation

The optimal configuration (*K* = 2.5, BQT = 15, and RQT = 15) using ${\text{PTT}}_{\text{median}\_\text{median}}$ achieved excellent accuracy with a mean difference of 0.25 ± 0.77 m/s compared to applanation tonometry. Detailed performance metrics under different configurations are presented in Table 1. Recordings that did not meet the minimum quality criteria—based on the BQT and RQT thresholds—were automatically excluded from the analysis. The number of subjects and recordings reported in Table 1 refer only to those retained after this quality filtering step.

**Table 1:** Mean and standard deviation of the difference between the automatic methods and the tonometry or manual method. *K* cross-correlation function; BQT number of peak pairs in the heartbeat; RQ: number of good heartbeats

Performance measure	*K*	*BQT*	*RQT*	Mean of difference (m/s)	Standard deviation of difference (m/s)	Number of patients	Number of recordings
Excellent	2.5	15	15	0.25	0.77	25	69
Acceptable	2.5	15	10	0.39	1.23	52	151

This approach ensures that the analysis is based on reliable signals, consistent with findings from a previous study [26], where only 15% of the measured signals were rated as good to excellent, making them immediately suitable for analysis.

Furthermore, to emphasize the advancements introduced by CAPE, we compared its performance against a previously published LDV-based method that was applied to the same dataset [25]. Unlike CAPE, the earlier method did not include any automatic signal quality assessment and depended on ECG-based detection. As shown in Table 2, CAPE achieved lower bias and standard deviation in cf-PWV estimation, highlighting its increased accuracy and robustness.

**Table 2:** Comparison of cf-PWV estimation accuracy between CAPE and the method by Badhwar et al. [25] on the same dataset

Method	Bias (m/s)	Standard deviation (m/s)	Key features
Badhwar et al. [25] with ECG	0.58	1.14	ECG used; no quality filtering
Badhwar et al. [25] without ECG	0.65	1.27	ECG-free; no quality filtering
CAPE (proposed method)	0.25	0.77	ECG-free; automatic quality assessment

**Fig. 7:**
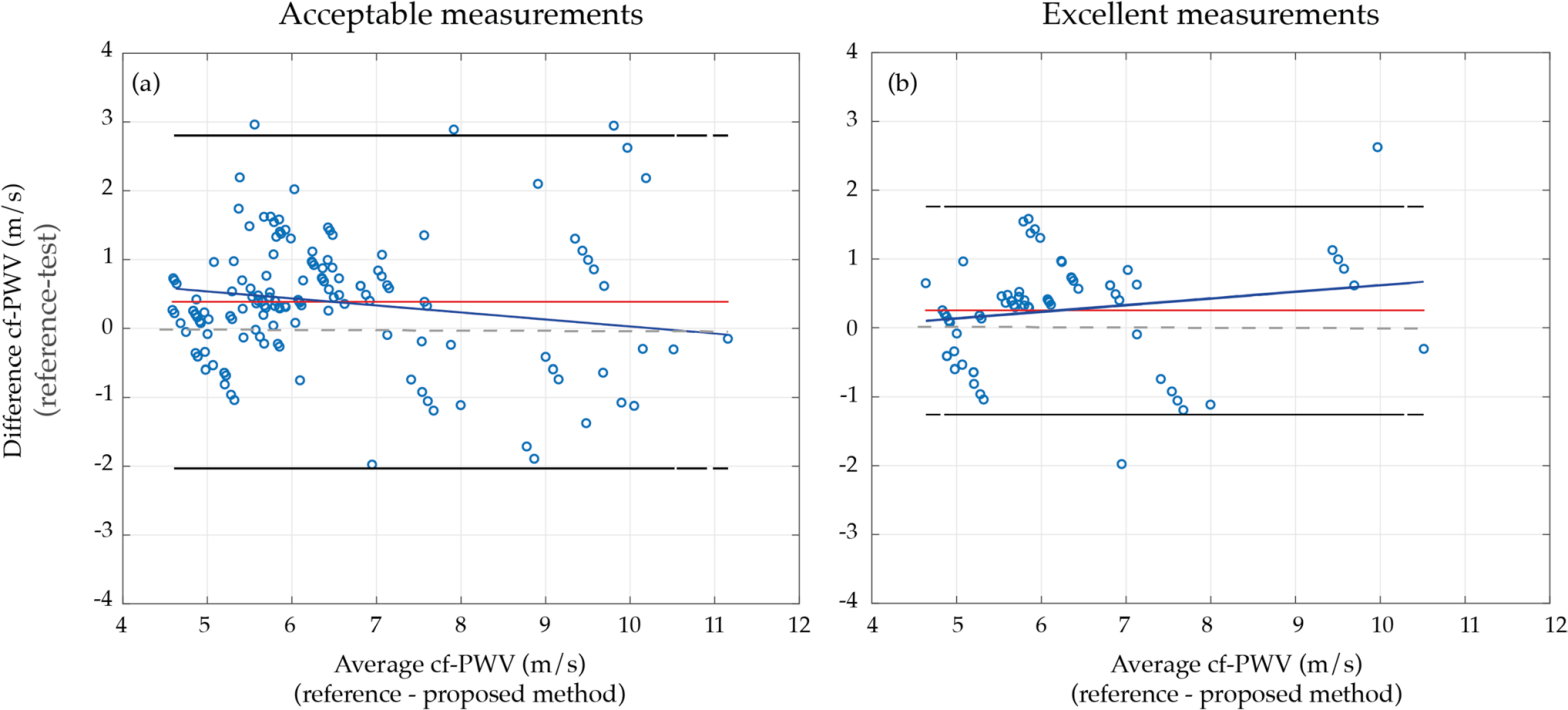
Bland–Altman analysis between the tonometry and the proposed algorithm, using the configuration for acceptable (**a**) and excellent (**b**) measurements. The red line represents the mean difference, while the black lines indicate the 95% limits of agreement, computed as the mean of difference ± 1.96 of the standard deviation difference. The grey dashed line represents zero

Bland–Altman analysis (Fig. 7) revealed a slight positive bias in both acceptable and excellent measurement configurations. For acceptable measurements, the bias was 0.39 ± 1.23 m/s (95% limits of agreement: − 2.02 to 2.80 m/s), while excellent measurements showed a bias of 0.25 ± 0.77 m/s (95% limits of agreement: − 1.26 to 1.76 m/s).

### Threshold tuning and ablation analysis

To determine the optimal values for the quality thresholds BQT and RQT, an empirical tuning procedure was conducted. Various combinations of these parameters were systematically tested across different values of the correction factor *K*. The configuration that achieved the best balance between estimation accuracy (measured in terms of mean difference and standard deviation relative to applanation tonometry) and the number of retained subjects and recordings was identified as *K* = 2.5, BQT = 15, and RQT = 15 (Table 3).

**Table 3:** Tuning threshold result: effects of the BQT, RQT, and *K* parameters on cf-PWV estimation accuracy. Values report the mean and standard deviation of the difference with applanation tonometry. *K* correlation threshold, *BQT* number of peak pairs, *RQT* number of high-quality beats. Bold values indicate the optimal trade-off between highest performance and the minimal number of discarded recordings

Performance measure	*K*	*BQT*	*RQT*	Mean of difference (m/s)	Standard deviation of difference (m/s)	Number of patients	Number of recordings
Acceptable	1	20	10	0.47	1.2003	52	154
Excellent	1	20	15	0.38	0.7043	26	58
Acceptable	2	20	10	0.32	1.2021	44	125
Excellent	2	20	15	0.33	0.6398	20	47
**Acceptable**	**2.5**	**15**	**10**	**0.39**	**1.23**	**52**	**151**
**Excellent**	**2.5**	**15**	**15**	**0.25**	**0.77**	**25**	**69**
Acceptable	3	15	10	0.27	1.20	37	102
Excellent	3	15	15	0.22	0.68	17	47
Acceptable	3.5	10	10	0.35	0.82	31	88
Excellent	3.5	10	15	0.16	0.70	17	49

The ablation analysis, summarized in Table 4, highlights the critical role of the BQT and RQT thresholds in ensuring high-quality PWV estimation. The BQT was disabled to assess how the inclusion of lower-quality beats influences overall PWV estimation. The results indicated a significant deterioration in the accuracy of PWV measurements, as a substantial number of low-quality beats were incorporated into the analysis, leading to increased variability and potential inaccuracies in the calculated PWV.

**Table 4:** Ablation study results: effects of BQT and RQT modules on cf-PWV estimations. *K* cross-correlation function, *RQT* number of peak pairs in the heartbeat, *RQT* number of good beats

*BQT*	*RQT*	Mean of difference (m/s)	Standard deviation of difference (m/s)
X	*✓*	2.56	2.39
*✓*	X	0.75	4.36
*✓*	*✓*	0.25	0.77

Additionally, the RQT was also disabled. This allowed recordings with insufficient valid beats to be considered in the analysis, negatively affecting the robustness of the PWV estimations. The data revealed that the absence of this threshold contributed to a marked increase in estimation error, particularly in recordings that were brief or contained excessive noise.

The findings from the ablation analysis underscore the necessity of implementing stringent quality control measures in cardiovascular signal analysis to enhance the reliability of PWV assessments.

The results obtained using the four approaches for computing the final delay, based on the module combination for excellent measurements (*K* = 2.5, BQT = 15, and RQT = 15), are summarized in Table 5, where ${\text{PTT}}_{\text{median}\_\text{median}}$ appears to be the most robust against outliers, displaying the smallest standard deviation computed between the LDV-based PTT estimation (performed with the four statistical approaches) and applanation tonometry.

**Table 5:** Comparison between LDV-based PTT estimation (performed over the entire recording) approaches and applanation tonometry. The best configuration is highlighted in bold

Estimation method	Mean of difference (m/s)	standard deviation of difference (m/s)
${\text{PTT}}_{\text{mean}\_\text{mean}}$	0.16	0.95
${\text{PTT}}_{\text{mean}\_\text{median}}$	− 0.25	2.23
${\text{PTT}}_{\text{median}\_\text{mean}}$	0.25	0.88
${\text{PTT}}_{\text{median}\_\text{median}}$	**0.25**	**0.77**

As an extension of our previous analysis on the same dataset [25], we investigated the relationship between cf-PWV values—measured using both LDV and applanation tonometry (AT)—and age, body mass index (BMI), and systolic blood pressure (SBP). For this analysis, Spearman’s correlation coefficients (*ρ*) and corresponding *p* values were computed for each modality. LDV-derived cf-PWV values were averaged per subject across all beats that met the “excellent” quality criterion (*n* = 25).

**Fig. 8:**
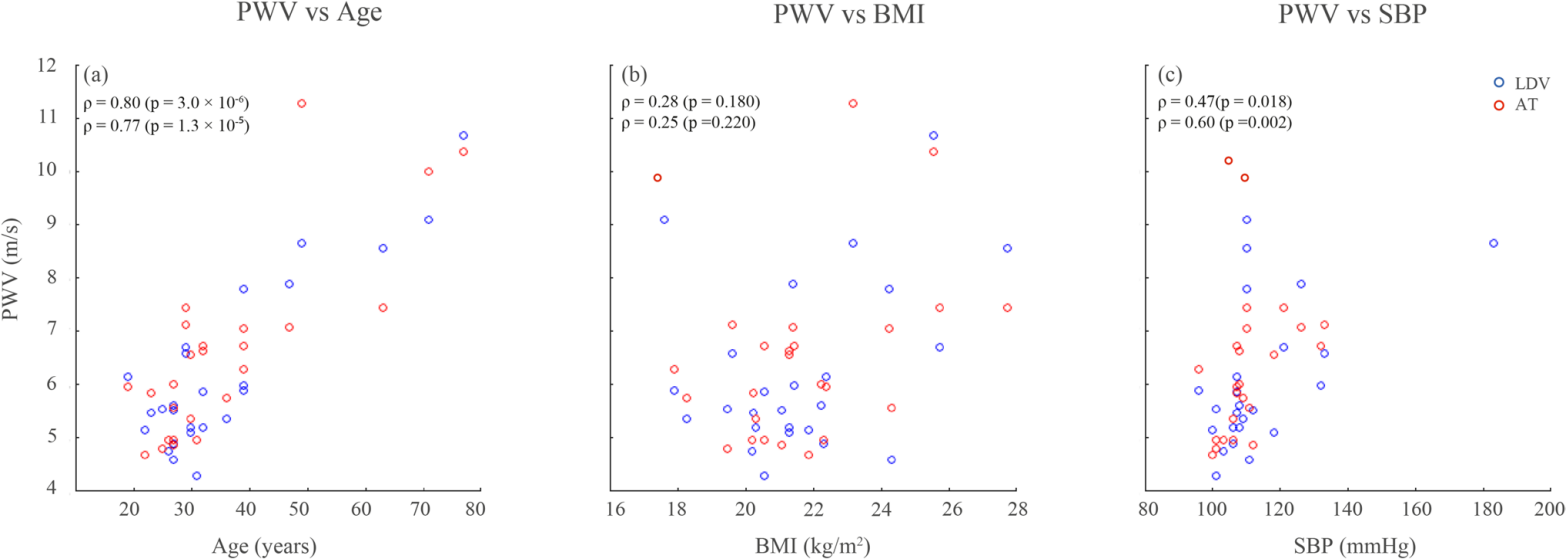
Scatterplots showing the association between cf-PWV values obtained from LDV (blue points) and applanation tonometry (AT, red points) with key physiological variables: **a** age (left); **b** body mass index—BMI; **c** systolic blood pressure—SBP

The scatterplots of cf-PWV versus age, BMI, and SBP for both LDV and AT modalities in Fig. 8 highlight that the two measurement methods exhibit similar associations with physiological quantities. In detail, cf-PWV_LDV showed strong correlation with age and moderate correlation with SBP, consistent with physiological expectations (and with AT measurements as well). Although weaker, correlations with BMI were comparable between modalities. Those findings further support the plausibility and potential generalizability of LDV-based cf-PWV estimation using the CAPE framework.

### Beat-to-beat analysis

**Fig. 9:**
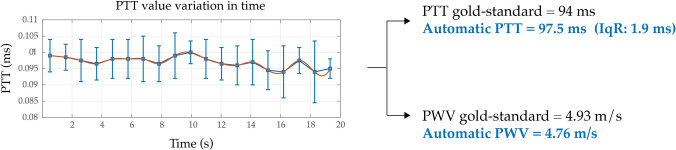
An example of PTT beat-to-beat variability with PTT and automatic PWV values from CAPE (blue) and applanation tonometry (black)

Finally, the proposed algorithm performs the beat-to-beat analysis. Figure 9 presents an example of the PTT variability on a beat-by-beat basis. In this context, variability refers to the global assessment of PTT fluctuations across all beats, rather than a direct difference between consecutive beats. By evaluating these fluctuations, it is possible to qualitatively assess the robustness of the measurement.

**Fig. 10:**
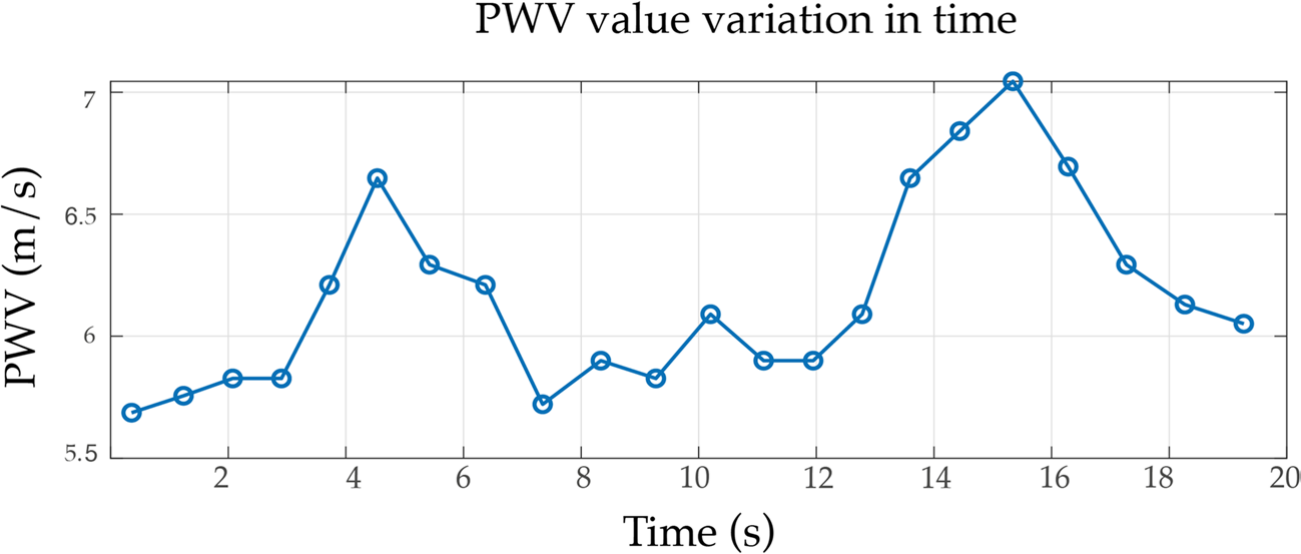
An example of the time-varying trend of cf-PWV. Beats that are not classified as of good quality are excluded, and the values in between are interpolated to maintain the continuity of the trend

As a final analysis, we present the time-varying trend of cf-PWV, as shown in Fig. 10. The results indicate that PWV exhibits beat-to-beat variability with a slow trend that could be ascribed to physiological lower frequency activity such as breathing and/or so-called Mayer waves [35]. Breathing could influence intrathoracic pressure and modulate arterial blood pressure, potentially leading to cyclic changes in transit time.

## Discussion

This study presents CAPE, a near real-time, accuracy-controlled framework for cf-PWV estimation using an LDV system. CAPE offers a continuous (beat-to-beat analysis) as well as an overall cf-PWV estimation, providing a comprehensive understanding of cardiovascular dynamics. To ensure accuracy, CAPE applies a quality criterion that assesses the reliability of measurements in advance, based on the quality of heartbeats and recordings. It is designed to handle variable quality single pulses (heartbeats) and use only those that meet established standards for the cf-PWV estimation, resulting in more reliable measurements. Indeed, by considering the number of cf-peak pairs (BQT) and the number of high-quality heartbeats (RQT), CAPE performs the cf-PWV estimation, with a specific confidence level, classifying it as excellent, acceptable, or unreliable. This *local–global* mechanism is critical in real-world conditions, where signals may be irregular or of suboptimal quality [26]. Furthermore, the dual-tuning architecture ensures flexibility, allowing CAPE to maintain performance across various signal recording levels and deliver either acceptable or high-accuracy measurements, based on data quality. CAPE is fully automated; once the signals are acquired, the analysis proceeds without any other operator intervention, minimizing operator dependency and improving reproducibility in clinical applications.

CAPE demonstrated excellent accuracy with improved performance compared to a previous LDV-based study which using the same dataset [25] exhibited a standard deviation of 1.27 m/s versus CAPE’s standard deviation of 0.77 m/s, when compared to applanation tonometry. These results underscore the importance of applying quality criteria to ensure the algorithm produces accurate measurements. However, the use of these criteria also led to a reduction in the analyzed dataset, as low-quality recordings were excluded from validation. Specifically, the sample size decreased to 25 patients when only measurements with an excellent confidence level were considered but increased to 52 patients with an acceptable confidence level. This consideration is in line with a previous study where a by-visual inspection quality assessment was implemented [26], and where only 15% of signals were rated from good to excellent, while 52% were classified as poor (20%) or suboptimal (32%).

Finally, CAPE’s ability to perform cf-PWV estimation within just 3 s of latency (tested on MacBook Pro 15, 2.6 GHz Intel Core i7 6 core) highlights its computational efficiency, making it an ideal candidate for integration into real LDV devices such as the CARDIS system. The preprocessing step, including acceleration calculation and signal filtering, takes approximately 1.7 s, while the quality assessment and fiducial point detection, along with PWV calculation, are completed in about 1.3 s. The clinical potential of our framework extends beyond standard PWV measurements. Its rapid processing capabilities make it suitable for continuous monitoring during surgical procedures, intensive care settings, and routine cardiovascular screening. The automated quality assessment feature could particularly benefit clinical research studies requiring large-scale arterial stiffness measurements, where operator expertise might vary across different centers. Another key strength of the proposed pipeline is its ability to estimate cf-PWV without relying on an ECG signal, which is typically used to detect and synchronize fiducial points [26]. By eliminating the need for ECG, the method simplifies the setup and reduces patient preparation time, all while preserving measurement accuracy. This makes it especially valuable in clinical settings where quick and efficient assessments are essential.

Despite the promising performance of CAPE, several limitations should be acknowledged. First, the thresholds used in the framework—such as BQT and RQT values—were determined using a single dataset (the CARDIS one). While these threshold values proved effective within this cohort, further validation is needed to assess their generalizability across diverse populations and measurement conditions, particularly in pathological cohorts such as the elderly, individuals with diabetes, or patients with advanced cardiovascular disease. Additionally, CAPE relies on template matching for fiducial point detection. Although this method performed well in the CARDIS cohort [26], it may be sensitive to variations in signal morphology. The framework’s *local–global* quality assessment enhances the reliability of cf-PWV estimation by filtering out poor-quality signals. However, this process also reduces the number of usable recordings, representing a trade-off between estimation accuracy and data availability. This trade-off should be taken into account when applying CAPE in real-world settings. Future studies should explore the robustness of the quality assessment approach, particularly in datasets characterized by lower signal quality or greater physiological heterogeneity.

Finally, the integration of machine learning or deep learning models [36] could further enhance CAPE’s adaptability by learning from signal variability, thus reducing the need for manual threshold tuning. These models could improve the algorithm’s robustness, especially in populations with challenging physiologies. Future work may also include the development of a measurement uncertainty parameter, based on signal quality metrics, to complement the final cf-PWV output.

## Conclusion

This study introduces the CAPE framework, a near real-time, accuracy-controlled system for cf-PWV estimation using LDV signals. CAPE provides continuous, beat-to-beat assessments of arterial stiffness, integrating quality control mechanisms to ensure reliable measurements across varying signal qualities. By evaluating the number of cf-peak pairs (BQT) and high-quality heartbeats (RQT), CAPE classifies measurements into confidence levels, adapting to real-world conditions. The integration of CAPE within the CARDIS device represents a significant advancement in cf-PWV estimation, offering an automated, near real-time solution for continuous cardiovascular monitoring and enhancing clinical assessments of arterial stiffness.
